# Use of renin-angiotensin system blockers and posttraumatic stress disorder risk in the UK Biobank: a retrospective cohort study

**DOI:** 10.1186/s12916-024-03704-5

**Published:** 2024-10-23

**Authors:** Sunghyuk Kang, Jimin Kim, Ji Su Yang, Ye Jin Jeon, Hyeok-Hee Lee, Shakira F. Suglia, Alexander C. Tsai, Jee In Kang, Sun Jae Jung

**Affiliations:** 1 Department of Preventive Medicine, Yonsei University College of Medicine, Yonsei-Ro 50-1, Seodaemun-Gu, Seoul, 03722 South Korea; 2 Department of Psychiatry and Institute of Behavioural Science in Medicine, Yonsei University College of Medicine, Seoul, Korea; 3 Department of Public Health, Graduate School, Yonsei University, Seoul, Korea; 4 Department of Internal Medicine, Yonsei University College of Medicine, Seoul, Korea; 5 Department of Epidemiology, Rollins School of Public Health, Emory University, Atlanta, GA USA; 6 Center for Global Health and Mongan Institute, Massachusetts General Hospital, Boston, MA USA; 7 Harvard Medical School, Boston, MA USA

**Keywords:** Antihypertensive agents, Angiotensin-converting enzyme inhibitors, Angiotensin receptor antagonists, Stress disorders, post-traumatic, Propensity score

## Abstract

**Background:**

Previous research has shown that the use of renin-angiotensin system (RAS) blockers is linked to a lower prevalence of posttraumatic stress disorder (PTSD), but longitudinal studies are scarce. We aimed to estimate the association between the use of RAS blockers and the risk of PTSD among individuals taking antihypertensive medications.

**Methods:**

This longitudinal study included participants aged 40–69 from the UK Biobank. Exposure data were obtained from the initial assessment (2006–10), while outcome data were obtained from the online mental health questionnaire administered 6–11 years later (2016–17). We included participants who were under antihypertensive treatment and did not have a prior diagnosis of PTSD before the initial assessment. Use of RAS blockers was defined as self-reported regular use, at the initial assessment, of angiotensin-converting enzyme inhibitor (ACEi) or angiotensin receptor blocker (ARB). Among participants who experienced adverse life experiences, cases of probable PTSD were defined with the six-item PTSD Checklist-Civilian version score ≥ 14. Logistic regression with inverse probability of treatment weighting was used to estimate the odds ratios (ORs) and 95% confidence interval (CI) for the association between RAS blocker use and the risk of probable PTSD.

**Results:**

Of the 15,954 participants (mean age = 59.9 years; 42.6% women) under antihypertensive treatment with no prior history of PTSD at the initial assessment, 64.5% were taking RAS blockers. After a mean follow-up of 7.5 years, 1,249 (7.8%) were newly identified with probable PTSD. RAS blocker users had a lower risk of probable PTSD than RAS blocker non-users (OR = 0.84 [95% CI: 0.75–0.94]), whereas the use of other antihypertensive medications showed no such association (users vs. non-users; calcium channel blockers, OR = 0.99 [95% CI: 0.88–1.11]; beta-blockers, 1.20 [1.08–1.34]; and thiazide-related diuretics, 1.15 [1.03–1.29]). The association between probable PTSD risk and the use of ACEi vs. ARB showed no significant difference (*p* = 0.96).

**Conclusions:**

Among individuals under antihypertensive treatment, the use of RAS blockers was associated with a decreased risk of probable PTSD. This added benefit of RAS blockers should be considered in the selection of antihypertensive medications.

**Supplementary Information:**

The online version contains supplementary material available at 10.1186/s12916-024-03704-5.

## Background

Posttraumatic stress disorder (PTSD) is a chronic mental disorder that affects a substantial number of individuals, with a lifetime prevalence of approximately 4–9% in U.S., U.K., and Canadian general population [1–5]. PTSD develops among individuals with a history of trauma and is characterized by symptoms such as re-experiencing the traumatic event, avoidance of trauma-related stimuli, negative alterations in cognition and mood, and hyperarousal [6]. Although various pharmacological and non-pharmacological interventions for PTSD have been developed, there remains a need to further prevent and manage PTSD to mitigate its burden on affected individuals’ lives [7, 8].

The renin-angiotensin system (RAS) has been implicated in the link between trauma and PTSD. Renin is released in response to stress, with plasma levels elevated among individuals exposed to trauma [9]. Severe stress sensitizes angiotensin II signaling in the central nervous system of rodents [10]. Moreover, individuals with PTSD have been shown to have altered RAS activities [9, 11]. Given these consistent strands of inquiry, modification of RAS could play a key role in the development of PTSD following trauma or, conversely, in the improvement of its symptoms.

RAS blockers, including angiotensin-converting enzyme (ACE) inhibitors and angiotensin receptor blockers (ARBs), are types of medications used to lower blood pressure by inducing vasodilation and inhibiting sodium retention. In addition to their protective effects on diabetic kidney diseases [12] and heart diseases [13, 14], RAS blockers have also been associated with decreased risks of mood disorders [15, 16]. Although the biological mechanisms underlying the putative neuroprotective effects of RAS blockers have not been fully elucidated, modulation of neuroinflammation [17], oxidative stress [18], the hypothalamic‒pituitary‒adrenal (HPA) axis [19], and the autonomic nervous system [20] are all thought to play a role. Additionally, RAS blockers may be associated with modifying conditioned fear memories [21, 22], a core pathology of PTSD [23].

Cross-sectional studies have suggested an association between RAS blocker use and a lower prevalence of PTSD [24, 25]. Evidence from longitudinal studies has been conflicting. A population-based cohort study in Denmark demonstrated that treatment with RAS blockers was not associated with lower incidence of PTSD [26]. In a randomized, placebo-controlled trial, authors found that the ARB losartan did not reduce PTSD symptoms over 10 weeks of follow-up [27]. To contribute to this conflicting literature, we investigated the association between use of RAS blockers and the risk of PTSD using a longitudinal cohort, the UK Biobank. We hypothesized that individuals using RAS blockers would have lower risk of developing PTSD than those who do not.

## Methods

### Data source

The UK Biobank is an on-going population-based prospective cohort study that aims to serve as a resource for research on genetic, environmental, and lifestyle factors influencing various middle-aged and late-life disorders [28, 29]. It associates extensive and accurate exposure measurement with comprehensive follow-up, and multiple health-related outcomes. More than 500,000 participants were recruited from the United Kingdom between 2006 and 2010. Individuals aged 40–69 years registered with the National Health Service were invited to participate. Information on sociodemographics, lifestyle factors, medical history, and physical measurements was obtained [30]. In addition, the International Classification of Diseases (ICD) codes for inpatient care were obtained through health record linkage. From July 2016 to July 2017, participants were invited to participate in an online mental health questionnaire (MHQ). Approximately 31% of the participants completed the MHQ, and data were collected for diagnosing common mental disorders and risk factors for mental disorders [31].

### Study population

**Fig. 1:**
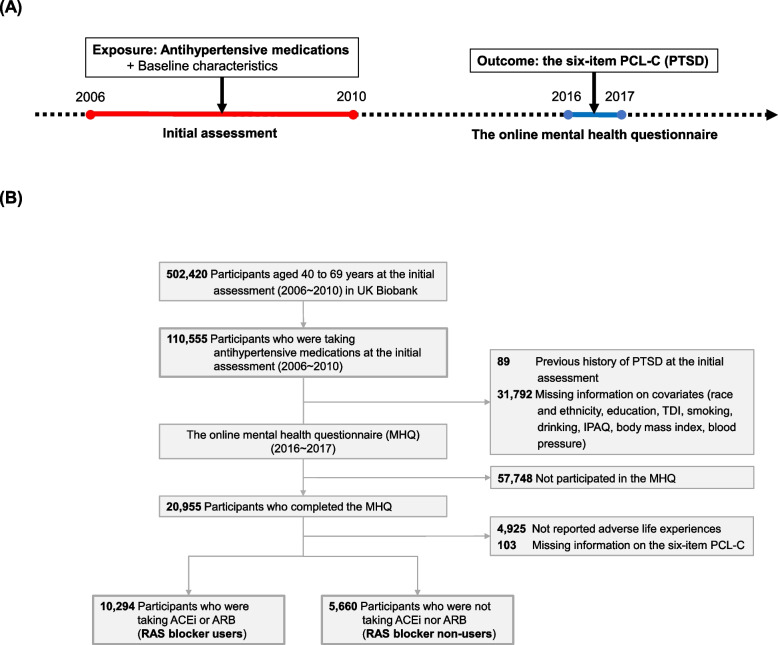
Graphical demonstrations of the study design. **A** Timeline of the UK Biobank study. **B** Flowchart of the study. RAS blockers include angiotensin-converting enzyme inhibitors and angiotensin receptor blockers. PTSD, posttraumatic stress disorder; MHQ, online mental health questionnaire; PCL-C, Posttraumatic Stress Disorder Checklist-Civilian Version; TDI, Townsend Deprivation Index; IPAQ, International Physical Activity Questionnaire; RAS, renin-angiotensin system

This retrospective cohort study included 502,420 participants enrolled in the initial assessment of the UK Biobank study between 2006 and 2010. Participants who reported taking antihypertensive medications at the initial assessment (*N* = 110,555) were selected. Participants with an inpatient PTSD diagnosis (ICD-10: F43.1, ICD-9: 309.81) or those who reported having been diagnosed with PTSD by a physician prior to the initial assessment were excluded (*N* = 89). Participants with incomplete information on the covariates were also excluded (*N* = 31,792). Participants who did not participate in the MHQ (*N* = 57,748), who did not report having experienced adverse life experiences (*N* = 4,925), and/or who were missing information on the outcome (*N* = 103) were excluded. To inquire about adverse life experiences in the MHQ, two screeners inquiring about catastrophic trauma (6 items) and adulthood adverse experience (5 items) were used (Additional file 1: Table S1) [31–33]. Participants who had experienced at least one adverse life experience were included [34]. A total of 15,954 participants were included in the analysis (Fig. 1).

### Exposure: RAS blockers

In the UK Biobank, trained nurses interviewed participants about their regular medication use [35]. Interviewers asked participants to provide names of all regular medications they were taking and retrieved each response from a list of generic and trade names of medications available in the UK. Any unlisted items were recorded as free text. Short-term medication use, such as a one-week course of antibiotics or medications previously taken but recently discontinued, was not recorded. Antihypertensive medications were identified using the Anatomical Therapeutic Chemical (ATC) codes (Additional file 2). Antihypertensive medications included ACE inhibitors, ARBs, beta-blockers, calcium channel blockers (CCBs), thiazide-related diuretics, and potassium-sparing diuretics. Combination formulations were considered multiple medications, each containing a single active ingredient.

Based on the self-report data obtained at the initial assessment, we classified participants taking antihypertensive medications into two groups: (1) Participants using RAS blockers (RAS blocker users) included participants taking only ACEi or ARB as well as those taking a RAS blocker in combination with any other antihypertensive medications (e.g., CCB). (2) Participants not taking RAS blockers (RAS blocker non-users) included those taking CCBs, beta-blockers, thiazide-related diuretics, and/or potassium-sparing diuretics. Antihypertensive combination therapy was defined as the use of more than one class of antihypertensive medications (RAS blockers, beta-blockers, CCBs, thiazide-related diuretics, and potassium-sparing diuretics).

### Outcome: probable PTSD

We defined probable PTSD using the six-item PTSD Checklist-Civilian Version (PCL-C) [31], a self-report questionnaire with each item scored on a five-point Likert scale (see Additional file 1: Table S1 for detailed information). Based on the Diagnostic and Statistical Manual of Mental Disorders (DSM)-IV Text-Revised, six items were constructed by selecting two highly correlated PCL-C items from each of the three symptom clusters: re-experiencing, avoidance and numbness, and hyperarousal. In the MHQ, the responses to the item on concentration difficulties were assessed using a four-point Likert-type scale, which constrained the range of the six-item PCL-C score to 6–29 (rather than 6–30). We defined probable PTSD as a score of ≥ 14, following the MHQ recommendation, as this cut-off value was shown to have 92% sensitivity and 78% specificity for PTSD diagnosed via a diagnostic interview [36, 37]. For the presence of specific PTSD symptoms, a score of ≥ 3 for each item was used as the cut-off [38].

### Covariates

Covariates included sex, age, socioeconomic status, lifestyle, and health-related factors. Self-reported racial and ethnic background was categorized as white (British, Irish, or any other white background) or non-white (mixed, Asian or Asian British, Black or Black British, Chinese, and other races). Educational status was ascertained based on whether participants had a college or university degree. The Townsend Deprivation Index (TDI) was calculated by aggregating data on car ownership, household overcrowding, owner occupation, and unemployment using participants’ postcodes [39]. We used the highest quartile of TDI scores among all UK Biobank participants to identify socioeconomically deprived individuals.

Smoking and drinking status were categorized as current, past, or never used. Physical activity level was classified as low, moderate, or vigorous based on the International Physical Activity Questionnaire category [40]. Overweight was defined as a body mass index (BMI) > 25 kg/m^2^. Participants who used antidepressants were identified using ATC codes (Additional file 3). History of diabetes, myocardial infarction (MI), and heart failure (HF) were defined as having an inpatient diagnosis prior to the initial assessment or reporting a previous physician diagnosis at the initial assessment. Uncontrolled hypertension was defined as a systolic blood pressure of ≥ 140 mmHg or a diastolic blood pressure of ≥ 90 mmHg in the initial assessment. Blood pressure was measured after 5 min of seated rest on two consecutive occasions with a 1-min interval using Omron 705 IT electronic blood pressure monitor (OMRON Healthcare) [41]. The mean of the first and second measurements was used. Detailed information and Unique Data Identifier for all variables are presented in Additional file 1: Table S2.

### Statistical analyses

We estimated the odds ratios (ORs) and 95% confidence intervals (CIs) for the association between RAS blocker use and probable PTSD using a logistic regression model. In order to establish a pseudo-population where unconditional exchangeability holds, we applied inverse probability weighting (IPW) [42]. We calculated weights for censoring to obtain exchangeability between included and excluded participants, as well as weights for treatment to obtain exchangeability between RAS blocker users and non-users. These two sets of weights were then multiplied and used as the final IPW weights. Age, sex, the interval between the initial assessment and the MHQ, race and ethnicity, educational status, smoking, drinking, BMI, antidepressant use, diabetes, MI, HF, uncontrolled hypertension, and antihypertensive combination therapy were used for calculating the inverse probability weights. Covariates were selected based on their known association with PTSD symptoms or antihypertensive medication use [43–45]. The covariate balance was defined as an absolute standardized difference < 0.1. Any covariate that remained unbalanced after IPW was additionally adjusted in the model.

Furthermore, we stratified our primary analyses by sex, age, and antihypertensive combination therapy. To extend the generalizability to the entire UK Biobank population, we conducted a secondary analysis by reincluding participants who were not taking antihypertensive medications.

We conducted five sensitivity analyses. First, we excluded participants with diabetes, MI, or HF, as these conditions are compelling indications for RAS blockers in addition to hypertension [46]. Excluding these participants would reduce the possibility of either structural or random zeroes undermining the positivity assumption [47, 48]. Second, we excluded participants with any history of mental disorders to enhance the homogeneity of the sample and minimize the possibility of confounding by unmeasured psychiatric morbidity. Participants with a history of mental disorders were identified on the basis of having an inpatient diagnosis of a mental disorder (ICD-10: F00-F99, ICD-9: 290–319) prior to the initial assessment or reporting at the initial assessment any physician diagnosis of a psychological/psychiatric problem (Additional file 1: Table S2). Third, we excluded beta-blockers from the definition of antihypertensive medications since they might be used primarily for psychotropic rather than for antihypertensive purposes [6]. Fourth, we re-estimated the models after re-defining probable PTSD as the simultaneous presence of the three symptom clusters, following the more stringent DSM-IV diagnostic algorithm as suggested by the original authors of the PTSD checklist [38]. Fifth, to alleviate influences of extreme weights, we re-estimated models after trimming IPW weights at the 1st and 99th, 5th and 95th, and 10th and 90th percentiles.

For a negative control analysis [49], we investigated the association between the use of other antihypertensive class (CCB, beta-blocker, and thiazide-related diuretics) and probable PTSD. We also separately estimated the risk of probable PTSD associated with the use of ACEi and ARB. We performed a complete case analysis, which is advantageous when the missingness cannot be assumed with certainty to be missing at random and is more likely to be missing not at random [50]. All analyses were conducted using SAS 9.4 (SAS Institute Inc., Cary, North Carolina, USA) and R software V.4.2.1 [51].

## Results

Among the 15,954 participants who were taking antihypertensive medications at baseline were 6,798 (42.6%) women, with a mean age (standard deviation [SD]) of 59.9 (6.4) years; nearly all of the cohort were white (15,419 [96.6%]). The distributions of adverse life experiences are shown in Supplementary Table 3. Notably, 10,294 (64.5%) used RAS blockers at baseline. Among the RAS blocker users, 2,413 (23.4%) also used CCBs, 2,220 (21.6%) also used beta-blockers, and 2,834 (27.5%) also used thiazide-related diuretics. Among the RAS blocker non-users, 2,111 (37.3%) used CCBs, 2,753 (48.6%) used beta-blockers, and 1,976 (34.9%) used thiazide-related diuretics. The baseline characteristics of the participants stratified according to RAS blocker use are shown in Table 1. The IPW weight distributions before and after stabilization are shown in Additional file 1: Fig. S1. Baseline covariate differences between excluded and included participants were balanced after weighting for censoring (Additional file 1: Table S4).

**Table 1:** Baseline characteristics of the participants

Variables	RAS blocker users (*N* = 10,294)	RAS blocker non-users (*N* = 5,660)	Absolute standardized difference^a^	
			**Before IPW**	**After IPW**
***At the initial assessment (2006 ~ 2010)***				
**Sex, Women, No. (%)**	3,773 (36.7)	3,025 (53.4)	**0.168**	0.003
**Age, Mean (SD)**	59.9 (6.4)	59.9 (6.5)	0.011	0.009
**Race and ethnicity, No. (%)**			0.009	0.005
White	9,981 (97.0)	5,438 (96.1)		
Non-white^b^	313 (3.0)	222 (3.9)		
**College or University degree, No. (%)**	4,159 (40.4)	2,174 (38.4)	0.020	0.002
**Socioeconomically deprived,**^**c**^** Q4, No. (%)**	2,189 (21.3)	1,241 (21.9)	0.007	0.007
**Smoking status, No. (%)**				
Never	4,902 (47.6)	2,887 (51.0)	0.034	0.007
Previous	4,755 (46.2)	2,404 (42.5)	0.037	0.006
Current	637 (6.2)	369 (6.5)	0.003	0.002
**Drinking status, No. (%)**				
Never	280 (2.7)	201 (3.6)	0.008	0.003
Previous	383 (3.7)	207 (3.7)	0.001	0.004
Current	9,631 (93.6)	5,252 (92.8)	0.008	0.007
**Physical activity (IPAQ), No. (%)**				
Low	2,308 (22.4)	1,140 (20.1)	0.023	0.001
Moderate	4,442 (43.2)	2,572 (45.4)	0.023	0.003
High	3,544 (34.4)	1,948 (34.4)	< 0.001	0.002
**BMI > 25 kg/m**^**2**^**, No. (%)**	8,454 (82.1)	4,278 (75.6)	0.065	0.008
**Antidepressants use, No. (%)**	810 (7.9)	593 (10.5)	0.026	0.005
**Diabetes, No. (%)**	1,707 (16.6)	322 (5.7)	**0.109**	0.026
**Myocardial infarction, No. (%)**	1,078 (10.5)	253 (4.5)	0.060	0.007
**Heart failure, No. (%)**	202 (2.0)	32 (0.6)	0.014	< 0.001
**Uncontrolled hypertension, No. (%)**	6,034 (58.6)	3,272 (57.8)	0.008	0.022
**Antihypertensive combination therapy, No. (%)**	5,946 (57.8)	1,209 (21.4)	**0.364**	0.005
**Antihypertensive medication class,**^**d**^** No. (%)**				
RAS blockers^e^	10,294 (100.0)	0 (0)	NA	NA
Calcium channel blockers	2,413 (23.4)	2,111 (37.3)	NA	NA
Beta-blockers	2,220 (21.6)	2,753 (48.6)	NA	NA
Thiazide-related diuretics	2,834 (27.5)	1,976 (34.9)	NA	NA
K^+^ sparing diuretics	102 (1.0)	140 (2.5)	NA	NA
***At the online mental health questionnaire (2016 ~ 2017)***				
**Probable PTSD,**^**f**^** No. (%)**	737 (7.2)	512 (9.0)	NA	NA
**The duration between the initial assessment and the online mental health questionnaire, year, Mean (SD)**	7.5 (0.8)	7.5 (0.8)	0.033	0.042

*IPW* Inverse probability weighting, *RAS* Renin-angiotensin system, *IPAQ* International Physical Activity Questionnaire, *BMI* Body mass index, *SD* Standard deviation, *PTSD* Posttraumatic stress disorder, *PCL-C* Posttraumatic stress disorder CheckList Civilian Version

^a^Sex, age, the time interval between assessments, race, education, TDI, smoking, drinking, physical activity, body mass index, antidepressants use, diabetes, myocardial infarction, heart failure, uncontrolled hypertension, and antihypertensive combination therapy were used in IPTW

^b^Non-White: Mixed, Asian or Asian British, Black or Black British, Chinese, and others

^c^Socioeconomically deprived individuals were defined as having a high score on Townsend Deprivation Index

^d^Duplicates exist due to antihypertensive combination therapy

^e^RAS blockers consist of angiotensin-converting enzyme inhibitors and angiotensin receptor blockers

^f^Probable PTSD: the six-item PCL-C score ≥ 14

Absolute standardized difference ≥ 0.1 was in bold

### Association between RAS blocker use and probable PTSD

**Fig. 2:**
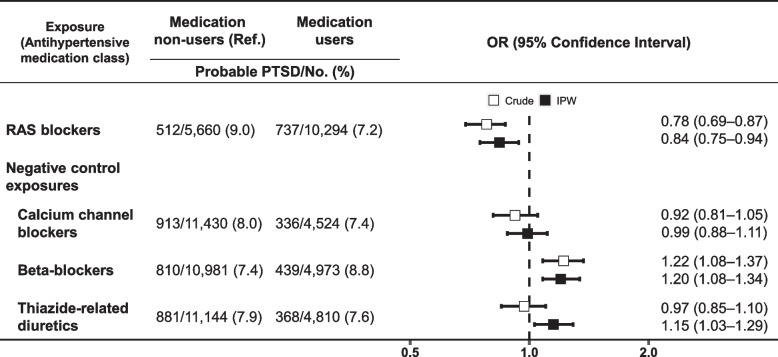
Associations between antihypertensive medications and PTSD. Sex, age, the time interval between assessments, race and ethnicity, education, TDI, smoking, drinking, physical activity, body mass index, antidepressant use, diabetes, myocardial infarction, heart failure, uncontrolled hypertension, and antihypertensive combination therapy were used in IPW. RAS blockers include angiotensin-converting enzyme inhibitors and angiotensin receptor blockers. Probable PTSD: the six-item PCL-C score ≥ 14. PTSD, posttraumatic stress disorder; OR, odds ratio; IPW, inverse probability treatment weighting; TDI, Townsend Deprivation Index; PCL-C, Posttraumatic Stress Disorder Checklist-Civilian Version; RAS, renin-angiotensin system

**Fig. 3:**
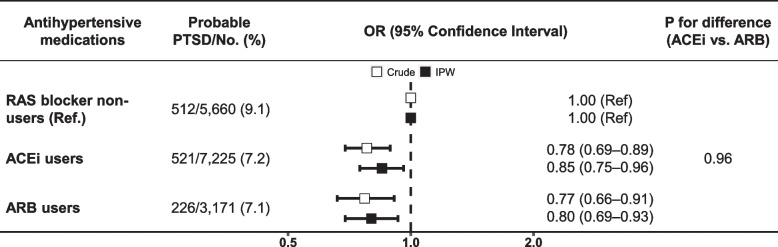
Associations of PTSD with ACEi and ARB. Sex, age, the time interval between assessments, race and ethnicity, education, TDI, smoking, drinking, physical activity, body mass index, antidepressants use, diabetes, myocardial infarction, heart failure, uncontrolled hypertension, and antihypertensive combination therapy were used in IPW. RAS blockers consist of angiotensin-converting enzyme inhibitors and angiotensin receptor blockers. Probable PTSD: the six-item PCL-C score ≥ 14. A total of 102 participants were taking both ACEi and ARB, and were consequently included in the group of ACEi users and ARB users; nevertheless, they were excluded from the analysis comparing ACEi users and ARB users. PTSD, posttraumatic stress disorder; RAS, renin-angiotensin system; ACEi, angiotensin-converting enzyme inhibitor; ARB, angiotensin receptor blocker; OR, odds ratio; IPW, inverse probability weighting; TDI, Townsend Deprivation Index; PCL-C, Posttraumatic Stress Disorder Checklist-Civilian Version

Total follow up time was 119,664 person-years, with each participant followed for a mean of 7.5 ± 0.8 (range: 5.9–10.2) years from the initial assessment. By follow-up, 1,249/15,954 (7.8%) participants were identified as having probable PTSD. Following application of the IPW, RAS blocker use was associated with a lower risk of probable PTSD (OR = 0.84 [95% CI: 0.75–0.94]) (Fig. 2). Other antihypertensive medication classes were not associated with a decreased risk of probable PTSD (users vs. non-users; CCBs, OR = 0.99 [95% CI: 0.88–1.11]). Use of beta-blockers or thiazide-related diuretics was paradoxically associated with an increased risk of probable PTSD (users vs. non-users; beta-blockers, OR = 1.20 [95% CI: 1.08–1.34]; thiazide-related diuretics, 1.15 [1.03–1.29]). We disaggregated the group of participants taking RAS blockers: the estimated associations between ACEi and probable PTSD (OR = 0.85 [95% CI: 0.75–0.96]), and between ARB and probable PTSD (0.80 [0.69–0.93]), were largely consistent with each other. No statistically significant difference was observed between ACEi and ARB users in their association with probable PTSD (ACEi vs. ARB, OR = 1.00 [95% CI: 0.86–1.18], p for difference = 0.96) (Fig. 3).

We conducted subgroup analyses to assess whether the associations differed by sex, age, or antihypertensive combination therapy. The associations between RAS blocker use and probable PTSD were generally consistent regardless of sex and age (Additional file 1: Table S5). However, this association was weaker in participants with antihypertensive combination therapy than in those with antihypertensive monotherapy (Monotherapy, OR = 0.77 [95% CI: 0.66–0.89]; combination therapy, 0.92 [0.77–1.10]; interaction-p = 0.023). We conducted the analysis covering the entire UK Biobank population rather than participants taking antihypertensive medications to extend the generalizability of our results. In these analyses, RAS blocker use was associated with a lower risk of probable PTSD (OR = 0.94 [95% CI: 0.90–0.99]), while use of other antihypertensive medications was associated with an increased risk of probable PTSD (users vs. non-users; CCBs, 1.21 [1.15–1.26]; beta-blockers, 1.33 [1.28–1.40]; thiazide-related diuretics, 1.15 [1.10–1.21]) (Additional file 1: Table S6).

The robustness of the primary analyses was confirmed through several sensitivity analyses (Additional file 1: Table S7). First, when participants with a history of diabetes, MI, or HF were excluded from the analyses, the association between RAS blocker use and probable PTSD remained statistically significant (OR = 0.81 [95% CI: 0.71–0.92]). Second, when participants with a history of mental disorders were excluded, the association was generally consistent with that in the primary analysis (OR = 0.82 [95% CI: 0.72–0.94]). Third, when beta-blockers were excluded from antihypertensive medications, the OR for the association between RAS blocker use and probable PTSD remained largely unchanged (OR = 0.87 [95% CI: 0.77–0.98]). Fourth, the association between RAS blocker use and probable PTSD following a more stringent DSM-based algorithm, the number of cases among exposed and unexposed declined but the statistically significant association remained (OR = 0.78 [95% CI: 0.66–0.93]). Fifth, in the repeated analyses using trimmed IPW weights, the associations were generally consistent with the primary analysis.

## Discussion

In this study of over 16,000 middle-aged adults under antihypertensive treatment participating in the UK Biobank, the regular use of RAS blockers was associated with a nearly two percentage point reduction in in the probability of probable PTSD (compared with adults using other antihypertensive medications) after a mean of 7.5 years of follow-up, or an approximately 16% decreased risk. Use of either ACEi and ARB was associated with a consistent lower risk of probable PTSD. The use of other antihypertensive medications was not associated with a decreased risk of probable PTSD. The associations between RAS blocker use and probable PTSD in subgroup and sensitivity analyses were generally consistent with those in the primary analyses.

Several previous studies have investigated the association between RAS blocker use and PTSD. In a cross-sectional study of 505 individuals from a clinic-based US cohort, participants who received ACEi or ARB had significantly fewer PTSD symptoms than those who did not [24]. Similarly, a cross-sectional study of 116,389 individuals from the biorepository database (The Partners Healthcare Biobank) in the US found that participants with PTSD had a significantly lower proportion of those taking ACEi or ARB compared to those without a PTSD diagnosis (29.4% vs. 32.4%) [25]. However, our findings were not consistent with the recently published study by Gradus et al. [26]. They analyzed a population-based cohort of trauma-exposed people in Denmark and found that CCB use was associated with a lower risk of PTSD, but the effect was imprecisely estimated (hazard ratio = 0.63 [95% CI: 0.34–1.20]), and neither ACEi (1.20 [0.67–2.10]) nor ARB (1.10 [0.48–2.40]) were associated with PTSD risk. Although the study by Gradus et al. included a large number of over 1.4 million individuals, random error might have produced the contrast between the use of CCB and ACEi/ARB due to the extreme low incidences of PTSD (0.06–0.13%), while we were able to provide robust estimates in multiple analyses. In addition, Gradus et al. adjusted for only Charlson comorbidity score, not for diabetes, MI, and HF, which are compelling indications for RAS blockers [46]. These conditions may be associated with a higher risk of PTSD, and MI specifically can lead to psychological trauma [52, 53]. If these conditions were not adjusted for, they may induce a spurious association between RAS blocker use and an increased risk of PTSD.

Our study extends the findings of previous studies in several ways. First, we confirmed the association between the use of RAS blocker and probable PTSD using a longitudinal design. By ensuring that the exposures preceded the outcomes, our design reduces, but does not eliminate, the possibility that the observed associations could be driven by reverse causality. Our study design should be interpreted as an intention-to-treat analysis, because we could not determine whether the participants maintained their antihypertensive regimen from the initial assessment to the MHQ [31]. Nonetheless, any discontinuation of RAS blockers during follow-up would have shifted the association toward null. Second, we specifically focused on those undergoing treatment for hypertension. It is suggested that individuals with hypertension are at a higher risk of developing PTSD due to factors such as shared pathophysiology and the stress associated with chronic illness [54–56]. Previous studies analyzed adults regardless of whether they were taking antihypertensive medications [24–26]. In an analysis not limited to people taking antihypertensives, the effect of RAS blocker would be conflated with the effect of treating hypertension. By restricting our study population to those taking antihypertensive medications, we could compare the average treatment effect of RAS blockers with the estimated counterfactual outcomes of taking RAS blockers [57].

RAS blockers influence several pathways within the central nervous system. The angiotensin II type 1 receptor, which ACEi and ARB inhibit, contributes to HPA axis activation, increased blood–brain barrier permeability, microglia activation, and the release of inflammatory cytokines [17–19, 58]. This broad neuroprotective effect of the RAS blockade underpins the association between RAS blocker use and the decreased risks of mood disorders and cognitive impairment [15, 16, 59]. More specifically to PTSD, previous preclinical studies showed that the administration of RAS blockers is associated with the fear extinction and reconsolidation in mice, suggesting fear modulation as a PTSD-specific mechanism of RAS blockade [21, 22]. Given that chronic administration of RAS blockers has been shown to enhance synaptic plasticity in the hippocampus, RAS blockers may impede the reduction in hippocampal function in patients with PTSD [60–62]. Additionally, the anti-inflammatory properties of RAS blockers may help reduce elevated levels of inflammatory cytokines in patients with PTSD [63–65]. However, whether alterations in biological pathways in individuals taking RAS blockers are associated with a lower risk of PTSD should be further studied to consolidate the causal association for PTSD.

In the present study, ACEi and ARB did not show differential associations with probable PTSD. However, in a previous cross-sectional study [25], ARB was associated with PTSD diagnosis, whereas ACEi was not. The superiority of ARB over ACEi for PTSD is reasonable, considering their mechanisms of action on angiotensin receptors. While both ACEi and ARB inhibit the neurotoxic angiotensin II type 1 receptor, only the former inhibits the neuroprotective angiotensin II type 2 receptor [66]. The possible advantage of ARB over ACEi in terms of preventing PTSD should be investigated in further studies.

In our study, beta-blocker users and thiazide-related diuretics users showed elevated ORs for probable PTSD compared to non-users. These results can be explained in several ways. Since the analyzed participants consisted of those taking antihypertensive medications, non-users of beta-blockers or diuretics had a higher proportion of RAS blocker use. Therefore, this reciprocal effect of RAS blockers might result in these findings. Additionally, the psychotropic properties of beta-blockers might confound the association by indication. However, this indication bias cannot explain the finding for thiazide-related diuretics. Lastly, beta-blockers and thiazide-related diuretics may have unfavorable effects on the development of PTSD, but further studies focusing on these medications are needed due to the scarcity of evidence to support this.

This study has several strengths. We obtained a large sample size from a well-defined, representative large-scale database, which enabled various subgroup and sensitivity analyses. Furthermore, we tried to include a large number of covariates to minimize the confounding of the association between RAS blocker use and probable PTSD. Despite these strengths, our study has several limitations. First, we defined probable PTSD using the six-item PCL-C rather than a diagnostic interview. However, as RAS blocker use is unlikely to affect the diagnostic process for PTSD, any potential misclassification would have been non-differential and shifted the associations towards null. We tried to mimic clinical diagnosis in the sensitivity analysis with alternative definition for probable PTSD, and the association seemed to be a little stronger (OR = 0.78 [95% CI: 0.66–0.93]). Second, baseline PTSD symptoms at the initial assessment were not considered. Although we excluded participants with a prior diagnosis of PTSD (*N* = 89), milder, undiagnosed cases of PTSD may not have been excluded. Therefore, prevalent cases of probable PTSD at baseline could be included in participants with probable PTSD at the MHQ, making the temporality of the association imperfect. Third, our results cannot be generalized to young individuals (less than 40 years old), non-British populations, or those with trauma exposures not measured in the UK Biobank. Specifically, since the age at onset of PTSD is mainly suggested to be in the 20 s or 30 s [1, 2], further studies focusing on young adults are needed. Fourth, self-reported medication use in the UK Biobank has not been validated. Further studies are needed to validate these self-reported data using external databases, such as linkage to primary case database. Fifth, the possibility of reverse causation cannot be ruled out. However, since no guidelines for hypertension incorporate psychiatric history into the treatment decision, the selection of antihypertensive medication should be considered “as randomized” with respect to any psychiatric morbidity.

## Conclusions

Regular use of RAS blockers was associated with a lower risk of probable PTSD after a mean follow-up of 7.5 years among middle-aged adults under antihypertensive treatment. The findings of this study may have implications for the choice of antihypertensive medications for the primary prevention of PTSD. This added benefit of RAS blockers should be considered in the selection of antihypertensive treatment, particularly for people at risk of trauma exposure and/or developing PTSD.

## Supplementary Information

Additional file 1: Fig. S1. – IPW weights before and after stabilization. Table S1-S2. – Descriptions of variables in UKB. Table S3. – Distributions of adverse life experiences. Table S4. – Characteristics of included and excluded populations. Table S5. – Subgroup analyses. Table S6. – Analysis reincluding participants not taking antihypertensive medications. Table S7. – Sensitivity analyses.

Additional file 2. List of included antihypertensive medications.

Additional file 3. List of included antidepressants.

## Data Availability

The data that support the findings of this study are available from the UK Biobank but restrictions apply to the availability of these data, which were used under license for the current study, and so are not publicly available. Data are, however, available from the authors upon reasonable request and with permission of the UK Biobank. More details can be found at https://www.ukbiobank.ac.uk.

## Abbreviations

PTSD: Posttraumatic stress disorder
RAS: Renin-angiotensin system
ACEi: Angiotensin-converting enzyme inhibitor
ARB: Angiotensin receptor blocker
HPA: Hypothalamic‒pituitary‒adrenal
ICD: International Classification of Diseases
MHQ: Online mental health questionnaire
ATC: Anatomical Therapeutic Chemical
CCB: Calcium channel blocker
PCL-C: Posttraumatic stress disorder Checklist-Civilian Version
DSM: Diagnostic and Statistical Manual of Mental Disorders
TDI: Townsend Deprivation Index
BMI: Body mass index
MI: Myocardial infarction
HF: Heart failure
OR: Odds ratio
CI: Confidence interval
IPW: Inverse probability weighting
SD: Standard deviation
