# Supplementary material for: Use of renin-angiotensin system blockers and posttraumatic stress disorder risk in the UK Biobank: a retrospective cohort study
Source: BMC Med. 2024 Oct 23;22:489. doi: 10.1186/s12916-024-03704-5 (PMC11515478; doi:10.1186/s12916-024-03704-5)
Supplement: Supplementary file 1 — Additional file 1: Fig. S1. – IPW weights before and after stabilization. Table S1-S2. – Descriptions of variables in UKB. Table S3. – Distributions of adverse life experiences. Table S4. – Characteristics of included and excluded populations. Table S5. – Subgroup analyses. Table S6. – Analysis reincluding participants not taking antihypertensive medications. Table S7. – Sensitivity analyses. [file 12916_2024_3704_MOESM1_ESM.docx]

**Additional File 1 Content**

Fig. S1. The distributions of IPW weights before and after stabilization

Table S1. Unique Data Identifier and descriptions of PTSD and trauma in UK Biobank study

Table S2. Unique Data Identifier and descriptions of key variables in UK Biobank study

Table S3. Distributions of adverse life experiences

Table S4. Comparison of baseline characteristics between included and excluded populations

Table S5. Subgroup analyses by sex, age, and antihypertensive combination therapy

Table S6. Analysis reincluding participants not taking antihypertensive medications

Table S7. Sensitivity analyses


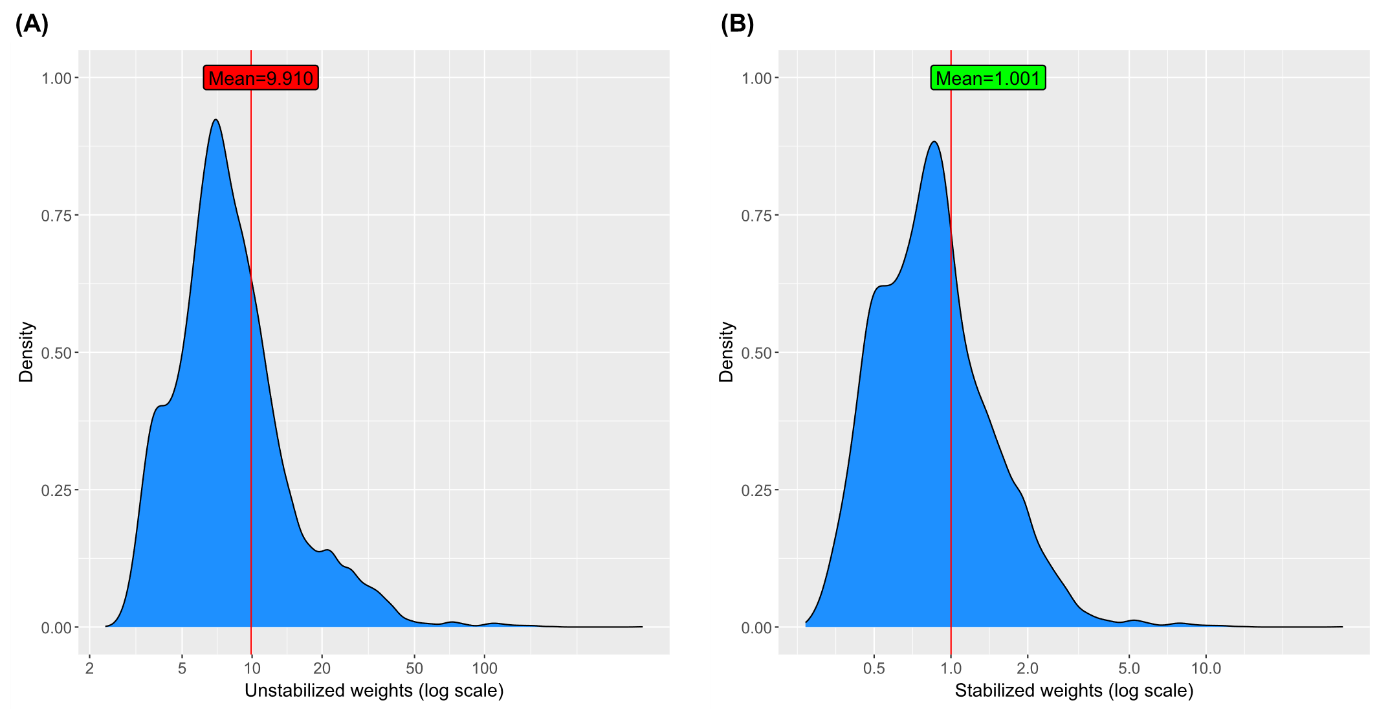


**Figure S1. The distribution of IPW weights before and after stabilization**

(A) The distribution of unstabilized weights. $W^{A,C}=W^{A}\times W^{C}=\left( \frac{1}{\Pr\left[ A | L \right]} \right)\times(\frac{1}{\Pr\left[ C=0 | L,A \right]})$(B) The distribution of stabilized weights. $SW^{A,C}=SW^{A}\times SW^{C}=\left( \frac{\Pr\left[ A \right]}{\Pr\left[ A | L \right]} \right)\times(\frac{\Pr\left[ C=0 | A \right]}{\Pr\left[ C=0 | L,A \right]})$ (A=exposure [RAS blocker use], L=covariates, C=indicator for censoring)

| **Table S1. Unique Data Identifier and descriptions of PTSD and trauma in UK Biobank study** | | |
| --- | --- | --- |
| **Variable** | **UDI in UKB** | **Description** |
| **The six-item PCL-C (probable PTSD)^1^** |  | The total score of the six-item PCL-C ranges from 6 to 29 and PTSD was defined as a score of 14 or higher. |
| Repeated disturbing thoughts of stressful experience in past month | 20497-0.0 | Five-point Likert scale (1–5): “Not at all” – “A little bit” – “Moderately” – “Quite a bit” – “Extremely”  Cut-off: ≥ 3 (“Moderately”, “Quite a bit”, and “Extremely”) |
| Felt very upset when reminded of stressful experience in past month | 20498-0.0 |  |
| Avoided activities or situations because of previous stressful experience in past month | 20495-0.0 |  |
| Felt distant from other people in past month | 20496-0.0 |  |
| Felt irritable or had angry outbursts in past month | 20494-0.0 |  |
| Recent trouble concentrating on things | 20508-0.0 | Four-point Likert scale (1–4): “Not at all” – “Several days” – “More than half the days” – “Nearly every day”  Cut-off: ≥ 3 (“More than half the days” and “Nearly every day”) |
| **Adult Trauma Screen (adulthood adverse experiences)** |  | Five-point Likert scale (1–5): “Never true” – “Rarely true” – “Sometimes true” – “Often” – “Very often true” |
| Interpersonal relationship problem | 20522-0.0 | Q: "Since I was sixteen... I have been in a confiding relationship"  Cut-off: ≤ 3 (“Never true”, “Rarely true”, and “Sometimes true”) |
| Physical abuse | 20523-0.0 | Q: "Since I was sixteen... A partner or ex-partner deliberately hit me or used violence in any other way"  Cut-off: ≥ 2 (“Rarely true”, “Sometimes true”, “Often”, and “Very often true”) |
| Emotional abuse | 20521-0.0 | Q: " Since I was sixteen... A partner or ex-partner repeatedly belittled me to the extent that I felt worthless"  Cut-off: ≥ 2 (“Rarely true”, “Sometimes true”, “Often”, and “Very often true”) |
| Sexual abuse | 20524-0.0 | Q: "Since I was sixteen... A partner or ex-partner sexually interfered with me, or forced me to have sex against my wishes"  Cut-off: ≥ 2 (“Rarely true”, “Sometimes true”, “Often”, and “Very often true”) |
| Financial problem | 20525-0.0 | Q: "Since I was sixteen... There was money to pay the rent or mortgage when I needed it"  Cut-off: ≤ 4 (“Never true”, “Rarely true”, “Sometimes true”, and “Often”) |
| **Catastrophic traumas** |  | Yes-no questions |
| Sexual assault | 20531-0.0 | Q: "In your life, have you...? Been a victim of a sexual assault, whether by a stranger or someone you knew" |
| Physically violent crime | 20529-0.0 | Q: "In your life, have you...? Been attacked, mugged, robbed, or been the victim of a physically violent crime" |
| Life-threatening accident | 20526-0.0 | Q: "In your life, have you...? Been in a serious accident that you believed to be life-threatening at the time" |
| Witnessed sudden violent death | 20530-0.0 | Q: "In your life, have you...? Witnessed a sudden violent death (eg. murder, suicide, aftermath of an accident)" |
| Life-threatening illness | 20528-0.0 | Q: "In your life, have you...? Been diagnosed with a life-threatening illness" |
| Combat/war-zone | 20527-0.0 | Q: "In your life, have you...? Been involved in combat or exposed to a war-zone (either in the military or as a civilian)" |
| ^1^ Lang AJ, Stein MB. An abbreviated PTSD checklist for use as a screening instrument in primary care. *Behaviour research and therapy* 2005;43(5):595-594.  UDI, Unique Data Identifier; PCL-C, Posttraumatic stress disorder Checklist-Civilian Version; PTSD, posttraumatic stress disorder | | |

| **Table S2. Unique Data Identifier and descriptions of key variables in UK Biobank study** | | |
| --- | --- | --- |
| **Variable** | **UDI in UKB** | **Description** |
| Date of attending assessment centre (the initial assessment) | 53-0.0 |  |
| Date of completing mental health questionnaire | 20400-0.0 |  |
| Sociodemographic factors |  |  |
| Sex | 31-0.0 |  |
| Age at the initial assessment | 21003-0.0 |  |
| Ethnic background (race) | 21000-0.0 |  |
| Qualifications (College or University degree) | 6138-0.x |  |
| Townsend Deprivation Index (socioeconomically deprived) | 189-0.0 | The highest quartile of TDI was defined for the entire population that participated in UK Biobank. |
| Lifestyle factors and anthropometric measurements |  |  |
| Smoking status | 20116-0.0 |  |
| Alcohol drinker status | 20117-0.0 |  |
| IPAQ activity group (physical activity) | 22032-0.0 |  |
| Body mass index | 21001-0.0 |  |
| Blood pressure | (Systolic) 4080-0.0, 4080-0.1  (Diastolic) 4079-0.0, 4079-0.1 | The means of the first and the second measurements were used. |
| Treatment/medication code | 20003-0.x | The self-reported uses of antihypertensive and antidepressant medications were identified using their ATC codes. |
| Information on in-patient diagnoses | (ICD-9)  Code: 41271-0.x  First date: 41281-0.x  (ICD-10)  Code: 41270-0.x  First date: 41280-0.x | The in-patient diagnoses recorded before the initial assessment date were used to identify participants who had diabetes (ICD-9; 250 / ICD-10; E10, E11, E12, E13, E14), myocardial infarction (ICD-9; 410, 411, 412 / ICD-10; I21, I22, I23), heart failure (ICD-9; 428 / ICD-10; I50), and PTSD (ICD-9; 30981 / ICD-10; F431). |
| Non-cancer illness code, self-reported | 20002-0.x | Diabetes: 1220 (diabetes), 1221 (gestational diabetes), 1222 (type 1 diabetes), and 1223 (type 2 diabetes)  Myocardial infarction: 1075 (heart attack/myocardial infarction)  Heart failure: 1076 (heart failure/pulmonary odema)  PTSD: 1469 (posttraumatic stress disorder)  Other psychiatric problems: 1286 (depression), 1291 (mania/bipolar disorder/manic depression), 1531 (post-natal depression), 1287 (anxiety/panic attacks), 1288 (nervous breakdown), 1289 (schizophrenia), 1290 (deliberate self-harm/suicide attempt), 1408-1410 (dependency), 1470 (anorexia/bulimia/other eating disorder), 1614 (stress), 1615 (ocd), 1616 (insomnia) |
| Medication for cholesterol, blood pressure, diabetes, or take exogenous hormones (self-report of antihypertensive medications) | (Men)6177-0.x  (Women)6153-0.x | Q: “Do you regularly take any of the following medications?”  A: “Cholesterol lowering medication”, “Blood pressure medication”, “Insulin”, “Hormone replacement therapy”, “Oral contraceptive pill or minipill” |
| Diabetes diagnosed by doctor | 2443-0.0 | Q: “Has a doctor ever told you that you have diabetes?” |
| UDI, Unique Data Identifier; ATC, Anatomical Therapeutic Chemical; ICD, International Classification of Diseases; PTSD, posttraumatic stress disorder | | |

| **Table S3. Distributions of adverse life experiences (N=15,954)** | |
| --- | --- |
| **Variable** | **No. (%)** |
| **Adult Trauma Screen (adulthood adverse experiences)** | 10,909 (68.4%) |
| Interpersonal relationship problem | 6,635 (41.6%) |
| Physical abuse | 2,189 (13.7%) |
| Emotional abuse | 4,296 (25.9%) |
| Sexual abuse | 882 (5.5%) |
| Financial problem | 3,323 (20.8%) |
| **Catastrophic traumas** | 11,432 (71.7%) |
| Sexual assault | 2,677 (16.8%) |
| Physically violent crime | 3,579 (22.4%) |
| Life-threatening accident | 2,128 (13.3%) |
| Witnessed sudden violent death | 3,075 (19.3%) |
| Life-threatening illness | 5,428 (34.0%) |
| Combat/war-zone | 973 (6.1%) |
| **Number of adverse life experiences (1~11)** |  |
| 1 | 6,236 (39.1%) |
| 2 | 4,684 (29.4%) |
| 3 | 2,524 (15.8%) |
| 4 | 1,340 (8.4%) |
| ≥5 | 1,170 (7.3%) |
|  | |

| **Table S4. Comparison of baseline characteristics between included and excluded populations** | | | | |
| --- | --- | --- | --- | --- |
| **Variables** | **Included**  **(N=15,954)** | **Excluded**  **(N=94,601)** | **Absolute standardized difference^a^** | |
|  |  |  | **Before IPCW** | **After IPCW** |
| **At the initial assessment (2006~2010)** | | | | |
| **Sex, Women, No. (%)** | 6,798 (42.6) | 44,995 (47.6) | 0.015 | 0.003 |
| **Age, Mean (SD)** | 59.9 (6.4) | 60.8 (6.5) | **0.139** | 0.018 |
| **Race and ethnicity, No. (%)** |  |  | 0.031 | <0.001 |
| White | 15,419 (96.6) | 87,880 (93.5) |  |  |
| Non-white^b^ | 535 (3.4) | 6,153 (6.5) |  |  |
| **College or University degree, No. (%)** | 6,333 (39.7) | 20,263 (22.0) | **0.160** | 0.003 |
| **Socioeconomically deprived,^c^ Q4, No. (%)** | 3,430 (21.5) | 26,937 (28.5) | 0.053 | 0.008 |
| **Smoking status, No. (%)** |  |  |  |  |
| Never | 7,789 (48.8) | 46,033 (49.0) | 0.003 | 0.001 |
| Previous | 7,159 (44.9) | 38,660 (41.2) | 0.029 | <0.001 |
| Current | 1,006 (6.3) | 9,157 (9.8) | 0.032 | 0.001 |
| **Drinking status, No. (%)** |  |  |  |  |
| Never | 481 (3.0) | 5,593 (5.9) | 0.024 | 0.001 |
| Previous | 590 (3.7) | 4,718 (5.0) | 0.011 | 0.001 |
| Current | 14,883 (93.3) | 83,951 (89.1) | 0.035 | 0.002 |
| **Physical activity (IPAQ), No. (%)** |  |  |  |  |
| Low | 3,448 (21.6) | 15,642 (22.5) | 0.007 | 0.001 |
| Moderate | 7,014 (44.0) | 28,665 (41.2) | 0.028 | 0.002 |
| High | 5,492 (34.4) | 25,235 (36.3) | 0.021 | 0.001 |
| **BMI >25, No. (%)** | 12,732 (79.8) | 77,441 (82.5) | 0.024 | 0.002 |
| **Antidepressants use, No. (%)** | 1,403 (8.8) | 10,201 (10.8) | 0.014 | 0.001 |
| **Diabetes, No. (%)** | 2,029 (12.7) | 15,759 (16.7) | 0.038 | 0.002 |
| **Myocardial infarction, No. (%)** | 1,331 (8.3) | 8,717 (9.2) | 0.011 | 0.002 |
| **Heart failure, No. (%)** | 234 (1.5) | 1,873 (2.0) | 0.005 | 0.001 |
| **Uncontrolled hypertension, No. (%)** | 9,306 (58.3) | 53,587 (60.9) | 0.024 | <0.001 |
| **Antihypertensive combination therapy, No. (%)** | 7,155 (44.8) | 44,948 (47.5) | 0.026 | 0.004 |
| **RAS blocker use, No. (%)** | 10,294 (64.5) | 60,271 (63.7) | 0.001 | 0.002 |
| ^a^Sex, age, race, education, TDI, smoking, drinking, physical activity, body mass index, antidepressants use, diabetes, myocardial infarction, heart failure, uncontrolled hypertension, and antihypertensive combination therapy were used in IPCW.  ^b^Non-White: Mixed, Asian or Asian British, Black or Black British, Chinese, and others  ^c^Socioeconomically deprived individuals were defined as having a high score on Townsend Deprivation Index.  Absolute standardized difference ≥0.1 was in bold.  IPCW, inverse probability of censoring weighting; IPAQ, International Physical Activity Questionnaire; BMI, body mass index; SD, standard deviation; | | | | |

| **Table S5. Subgroup analyses by sex, age, and antihypertensive combination therapy** | | | | | | |
| --- | --- | --- | --- | --- | --- | --- |
| **Subgroup** | **RAS blocker non-users (Ref.)** | **RAS blocker users** | **Crude model** | | **IPW** | |
|  | **Probable PTSD/No. (%)** | | **OR** | **(95% CI)** | **OR** | **(95% CI)** |
| **Primary analysis** | 512/5,660 (9.0) | 737/10,294 (7.2) | 0.78 | (0.69–0.87) | 0.84 | (0.75–0.94) |
| **Sex** | | | | | | |
| **Men** | 191/2,635 (7.2) | 404/6,521 (6.2) | 0.85 | (0.71–1.01) | 0.87 | (0.74–1.03) |
| **Women** | 321/3,025 (10.6) | 333/3,773 (8.8) | 0.82 | (0.69–0.96) | 0.80 | (0.69–0.95) |
| Interaction-p |  |  |  | | 0.669 | |
| **Age** | | | | | | |
| **Age <60** | 295/2,192 (13.5) | 421/4,185 (10.1) | 0.72 | (0.61–0.84) | 0.81 | (0.69–0.94) |
| **Age ≥60** | 217/3,468 (6.3) | 316/6,109 (5.2) | 0.82 | (0.68–0.98) | 0.90 | (0.76–1.06) |
| Interaction-p |  |  |  | | 0.169 | |
| **Antihypertensive combination therapy** | | | | | | |
| **Monotherapy** | 418/4,451 (9.4) | 315/4,348 (7.2) | 0.75 | (0.65–0.88) | 0.77 | (0.66–0.89) |
| **Combination therapy** | 94/1,209 (7.8) | 422/5,946 (7.1) | 0.91 | (0.72–1.14) | 0.92 | (0.77–1.10) |
| Interaction-p |  |  |  | | 0.023 | |
| RAS blockers consist of angiotensin-converting enzyme inhibitors and angiotensin receptor blockers.  Probable PTSD was defined as the six-item PCL-C score ≥14  Sex, age, the time interval between assessments, race, education, TDI, smoking, drinking, physical activity, body mass index, antidepressants use, diabetes, myocardial infarction, heart failure, uncontrolled hypertension, and antihypertensive combination therapy were used in IPW.  Variables used in weighting were all balanced. (Absolute standardized difference <0.1)  PTSD, posttraumatic stress disorder; IPW, inverse probability weighting; OR, odds ratio; CI, confidence interval; ACEi, angiotensin converting enzyme inhibitor; ARB, angiotensin receptor blocker | | | | | | |

| **Table S6. Analysis reincluding participants not taking antihypertensive medications** | | | | | | |
| --- | --- | --- | --- | --- | --- | --- |
| **Exposure (Antihypertensive medication class)** | **Medication non-users (Ref.)** | **Medication users** | **Crude model** | | **IPW** | |
|  | **Probable PTSD/No. (%)** | | **OR** | **(95% CI)** | **OR** | **(95% CI)** |
| **RAS blockers** | 6,583/83,131 (7.9) | 737/10,294 (7.2) | 0.90 | (0.84–0.97) | 0.94 | (0.90–0.99) |
| **Calcium channel blockers**^a^ | 6,984/88,901 (7.9) | 336/4,524 (7.4) | 0.94 | (0.84–1.05) | 1.21 | (1.15–1.26) |
| **Beta-blockers** | 6,881/88,452 (7.8) | 439/4,973 (8.8) | 1.15 | (1.04–1.27) | 1.33 | (1.28–1.40) |
| **Thiazide-related diuretics**^a^ | 6,952/88,615 (7.8) | 368/4,810 (7.7) | 0.97 | (0.87–1.09) | 1.15 | (1.10–1.21) |
| RAS blockers consist of angiotensin-converting enzyme inhibitors and angiotensin receptor blockers.  Probable PTSD was defined as the six-item PCL-C score ≥14  Sex, age, the time interval between assessments, race, education, TDI, smoking, drinking, physical activity, body mass index, antidepressants use, diabetes, myocardial infarction, and heart failure were used in IPW.  Unbalanced variables after weighting were additionally adjusted in logistic regression models.  ^a^Age was additionally adjusted in the logistic regression model with IPW  PTSD, posttraumatic stress disorder; IPW, inverse probability weighting; OR, odds ratio; CI, confidence interval; ACEi, angiotensin converting enzyme inhibitor; ARB, angiotensin receptor blocker | | | | | | |

| **Table S7. Sensitivity analyses** | | | | | | |
| --- | --- | --- | --- | --- | --- | --- |
| **Sensitivity analyses** | **RAS blocker non-users (Ref.)** | **RAS blocker users** | **Crude model** | | **IPW** | |
|  | **Probable PTSD/No. (%)** | | **OR** | **(95% CI)** | **OR** | **(95% CI)** |
| **Primary analysis** | 512/5,660 (9.0) | 737/10,294 (7.2) | 0.78 | (0.69–0.87) | 0.84 | (0.75–0.94) |
| **Excluding participants with diabetes, MI, and HF** | 447/5,096 (8.8) | 503/7,620 (6.6) | 0.74 | (0.64–0.84) | 0.81 | (0.71–0.92) |
| **Excluding participants with a history of mental disorders** | 350/5,050 (6.9) | 541/9,557 (5.7) | 0.81 | (0.70–0.93) | 0.82 | (0.72–0.94) |
| **Excluding beta-blockers from antihypertensive medications** | 291/3,638 (8.0) | 737/10,294 (7.2) | 0.89 | (0.77–1.02) | 0.87 | (0.77–0.98) |
| **Probable PTSD defined as presences of re-experiencing, avoidance and numbness, and hyperarousal symptoms** | 198/5,660 (3.5) | 299/10,294 (2.9) | 0.83 | (0.69–0.99) | 0.78 | (0.66–0.93) |
| **Trimming IPW weights** |  |  |  |  |  |  |
| **1^st^ and 99^th^ percentiles** |  |  |  |  | 0.82 | (0.73–0.92) |
| **5^th^ and 95^th^ percentiles** |  |  |  |  | 0.81 | (0.72–0.91) |
| **10^th^ and 90^th^ percentiles** |  |  |  |  | 0.80 | (0.71–0.91) |
| RAS blockers consist of angiotensin-converting enzyme inhibitors and angiotensin receptor blockers.  Probable PTSD was defined as the six-item PCL-C score ≥14  Sex, age, the time interval between assessments, race, education, TDI, smoking, drinking, physical activity, body mass index, antidepressants use, diabetes, myocardial infarction, heart failure, and antihypertensive combination therapy were used in IPW.  Variables used in weighting were all balanced. (Absolute standardized difference <0.1)  PTSD, posttraumatic stress disorder; IPW, inverse probability weighting; OR, odds ratio; CI, confidence interval; ACEi, angiotensin converting enzyme inhibitor; ARB, angiotensin receptor blocker | | | | | | |
